# Engaging a person with lived experience of mental illness in a collaborative care model feasibility study

**DOI:** 10.1186/s40900-020-00247-w

**Published:** 2021-01-08

**Authors:** Lenka Vojtila, Iqra Ashfaq, Augustina Ampofo, Danielle Dawson, Peter Selby

**Affiliations:** 1 Nicotine Dependence Clinic, Centre for Addiction and Mental Health, 175 College Street, Toronto, ON M5T1P Canada; 2 Addictions Research Program, Centre for Addiction and Mental Health, Toronto, Canada; 3 Department of Family and Community Medicine; Department of Psychiatry; Dalla Lana School of Public Health, University of Toronto, Toronto, Canada

**Keywords:** Collaborative care, People with lived experience, Peer engagement, Technology, Mental health

## Abstract

**Plain English summary:**

Researchers have explored different types of treatment to help people with a mental illness with other problems they might be experiencing, such as their health condition and quality of life. Care models that involve many different health care providers working together to provide complete physical and mental health care are becoming popular. There has been a push from the research community to understand the value of including people with lived experience in such programs. While research suggests that people with lived experience may help a patient’s treatment, there is little evidence on including them in a team based program. This paper describes how our research team included a person with lived experience of psychosis in both the research and care process. We list some guiding principles we used to work through some of the common challenges that are mentioned in research. Lastly, experiences from the research team, lessons learned, and a personal statement from the person with lived experience (AA) are provided to help future researchers and people with lived experience collaborate in research and healthcare.

**Abstract:**

**Background**

In our current healthcare system, people with a mental illness experience poorer physical health and early mortality in part due to the inconsistent collaboration between primary care and specialized mental health care. In efforts to bridge this gap, hospitals and primary care settings have begun to take an integrated approach to care by implementing collaborative care models to treat a variety of conditions in the past decade. The collaborative care model addresses common barriers to treatment, such as geographical distance and lack of individualized, evidence-based, measurement-based treatment. Person(s) with lived experience (PWLE) are regarded as ‘*experts by experience’* in the scope of their first-hand experience with a diagnosis or health condition. Research suggests that including PWLE in a patient’s care and treatment has significant contributions to the patient’s treatment and overall outcome. However, there is minimal evidence of including PWLE in collaborative care models. This paper describes the inclusion of a PWLE in a research study and collaborative care team for youth with early psychosis.

**Aims**

To discuss the active involvement of a PWLE on the research and collaborative care team and to describe the research team’s experiences and perspectives to facilitate future collaborations.

**Method**

This paper describes the inclusion of a PWLE on our research team. We provide a selective review of the literature on several global initiatives of including PWLE in different facets of the healthcare system. Additionally, we outline multiple challenges of involving PWLE in research and service delivery. Examples are provided on how recruitment and involvement was facilitated, with the guidance of several principles. Lastly, we have included a narrative note from the PWLE included in our study, who is also a contributing author to this paper (AA), where she comments on her experience in the research study.

**Conclusion**

Including PWLE in active roles in research studies and collaborative care teams can enhance the experience of the researchers, collaborative care team members, and PWLE. We showcase our method to empower other researchers and service providers to continue to seek guidance from PWLE to provide more comprehensive, collaborative care with better health outcomes for the patient, and a more satisfying care experience for the provider.

## Background

Individuals with mental illness have significantly higher rates of physical disorders than the general population, [1] due to a heightened risk of physical illness and reduced access to healthcare [2]. Several approaches have been used to address the discrepancy in health condition and quality of life among individuals with mental illness. For patients, collaborative care provides more access to treatment, reducing use of general medical services, increased mental health symptom management, and enhanced experiences of receiving care [3–5].

The collaborative care model (CCM) addresses common barriers in healthcare, including those within the mental health field. The CCM involves an ongoing relationship between specialized clinicians that provide comprehensive care for patients. Clinicians involved may include a care manager, nurse practitioner, family physician, and one or more specialists(s) (e.g., psychiatrist, endocrinologist, etc.). Quality Indicators for Collaborative Care, a Canadian-based collaborative care project, recommend including people with lived experience (PWLE) to play key roles in evaluating and improving CCMs [6]. Within the Collaborative Mental Health Care model, primary care and mental health care providers share resources, expertise and decision-making for more effective and coordinated mental health care [7]. The CCM can be related to the Asset Based Community Development (ABCD) approach, where the model’s principles focus on community development using the unique abilities of each contributor (e.g., communities, groups, residents) [8]. Both models focus on individual attributes that can collectively contribute to the progress and development of an outcome – whether it is a community, or an individual’s treatment. As such, the CCM model has a strong impetus to partner with patients and their families to facilitate direct patient care, service planning, evaluation, quality improvement and policy development [9].

PWLE are considered *experts by experience* within their diagnosis or health condition [10, 11]. “Nothing About Us Without Us” is a slogan commonly used to communicate that policies should not be made without full participation of members from the affected groups [12]. Using their own experience as knowledge, PWLE have many roles in the mental health community, including being a decision-maker in their own care. They may also be navigators in the mental health system, providing advocacy and empowerment as supportive workers. Lastly, PWLE may hold leadership roles in social policy, treatment development, and education [13, 14], however, such leadership roles are lacking in research and healthcare [15]. In the ABCD approach, these leadership roles are more prevalent through its implementation of residents and local assets to address community development. This approach has been implemented in healthcare setting, by affected individuals to aid in addressing and social determinants of health and identyifying common gaps in treatment delivery and health inequalities [8]. Initiatives among researchers, professional organizations and governments have explored including PWLE as active members in research and in the treatment for other patients that also have the same condition. Collaboration with adult PWLE increases the engagement and health outcomes among participants [16]. Similar findings have been found in peer engagement for youth program development [17]. Among adults, patient involvement in mental health and substance use settings reduced hospital readmission, increased patient well-being and engagement, and provided educational opportunities to health care staff and systems [18–26]. Lastly, several studies identified the importance of developing new processes of patient-oriented care, with the help of PWLE, rather than using existing practices with the hope that patients will adapt [27, 28].

Although the significance of including PWLE in mental healthcare has been established, their involvement in treatment, research, and policy has been minimal when compared to other health conditions [27]. In Canada, this gap is being recognized. The College of Family Physicians of Canada reported that PWLE should be included in primary care and educational settings to competently treat patients in mental healthcare [5]. The Public Health Agency of Canada announced the need for global collective action on mental health from all sectors of society, including PWLE, to address complex mental illness [29]. Mental Health Research Canada vows to improve the lives of individuals living with mental illness by incorporating PWLE and other providers to inform their care. For many research programs and grants in North America, the inclusion patient engagement is now a common requirement [30].

Other countries have also adopted models of including PWLE into research and intervention practices. For example, in Australia, the Queensland Mental Health, Drug and Alcohol Strategic Plan (2014–19) has committed to supporting the active engagement and leadership of PWLE in the mental health system, in policy, legislation, programs design, and service delivery [31]. By 2024, Australia plans to employ PWLE to a range of integrated and accessible health services [32]. The Mental Health Foundation of New Zealand released a position paper, with the leadership and advocacy of PWLE, to address New Zealand’s mental health system [33]. In the UK, government funding policy recommends involving PWLE in the National Health Service [34]. However, while encouraged, barriers to and effects of involving PWLE have not been evaluated extensively. In other countries, such as in certain areas of Asia, including PWLE in the mental health field is still minimal [35].

Involving PWLE in research can increase the appropriateness and quality of interventions, thereby improving future healthcare services [36]. The voices of PWLE have impacted mental health care more than ever before, by increasing awareness, reducing stigmatization, improving access to treatment and services, and providing valuable support [37]. This article is a descriptive extension of our study, focusing on the involvement of a PWLE and the research team’s experiences. This paper begins by presenting the original research study conducted and describing how a PWLE was included. Next, we identify several guiding principles used to ensure essential and purposeful engagement and collaboration. Lastly, we included a narrative note from the PWLE included in our study, who is also a contributing author to this paper (AA).

### Including a person with lived experience on the research and collaborative care team

Our study, Technology-Enabled Collaborative Care for Youth (TECC-Y), was a 12-week pragmatic randomized trial, testing the feasibility of our technology-enabled collaborative care (TECC) intervention to promote health behavior change in youth (ages 16–29) with early psychosis, using an e-platform. We recruited 70 participants, of which half received 1:1 sessions with a health coach who utilized motivational interviewing techniques to facilitate behaviour change (i.e., in areas of nutrition, physical activity and smoking cessation). Health coaches would discuss their caseload weekly with the virtual care team (VCT), consisting of a psychiatrist, addiction specialist, recreation therapist, nutritionist, dietitian, and a PWLE of psychosis. Our CCM was unique, as all interactions with the participants and between clinicians were held remotely via technology. Therein, personalized treatment recommendations were discussed virtually for areas including, but not limited to, medication adherence, mental health symptoms, and resources to support behavior change. This type of integrated care model was based on the National Institute of Mental Health’s NAVIGATE program, which had demonstrated successes in clinical and functional health outcomes using a comprehensive treatment approach for first-episode psychosis in the United States [38]. For more details on our study and treatment intervention, refer to the study protocol [39].

In the TECC-Y study, the aim in including a PWLE of psychosis on the research and VCT was to develop a model and intervention that would be relevant and empowering for participants with psychosis. Disempowerment and loss of confidence are common among individuals with early psychosis [40]. Our study approaches, i.e. client-centered, incorporating a PWLE on the study teams, and having a PWLE collaborative directly with the participants, was used to help decrease the authoritative relationship between participant and healthcare provider and increase participant empowerment. Similarly, this supportive role may in turn increase the empowerment of the PWLE [41]. As such, the research team collaborated with Augustina Ampofo (AA), a PWLE of psychosis, to involve her as an active member of the research team and the VCT. The participants were aware that there was a PWLE on the VCT, and AA’s role was to emphasize participant voices through her own personal accounts with psychosis. As such, AA’s dual role in the study was to provide this perspective as a member of the research team and the VCT. Her purpose was to help shape and individualize the research study and treatment recommendations based on her own experience with psychosis.

The research team did not have any selection criteria when recruiting a PWLE of psychosis, beyond clinical stability. Though prior research experience is always helpful, it was not necessary for our research study. A call for PWLE of psychosis was sent out by the Youth Engagement Initiative at the McCain Centre at CAMH in Toronto, Canada. AA was approached by the research team at a conference and was explained the purpose of the study and the team’s interest in her involvement. After the initial discussion, AA decided to join. A formal employment contract was signed with her job title being ‘Virtual Care Team (VCT) Member’, though was applicable for both research and clinical roles. The roles and responsibilities were outlined in the contract and remained flexible to change throughout the study period; these roles were monitored and adapted on an ongoing basis after check-in meetings. AA joined the team with a diverse background in research and her own personal experience in mental healthcare. AA played two active roles in the study. At the start of her participation, AA focused on her research role, which tasks included reviewing and editing the study protocol and ethics submissions, providing input into the design and development of the study e-platform, participating in research meetings, and providing feedback on any research-related issues (e.g., study flow). Additionally, AA co-facilitated a conference workshop and contributed to preparing a manuscript related to the study.

In AA’s second role as a PWLE on the VCT, she collaborated in participant case reviews, treatment planning, and provided input on the participant’s care with the clinical team. This included weekly meetings with the VCT, where her input was encouraged when creating treatment recommendations. She hosted live webinars for participants, sharing her experience with psychosis and allowing participants to share their own. Lastly, she participated as an online discussion board moderator, further supporting participants to make healthy changes in their lives.

### Initiatives and guiding principles

The research literature reports on the common challenges and barriers of including PWLE in research and treatment settings, such as lack of research training in methods and related tasks, ongoing supervision and management, tokenistic involvements and power dynamics (e.g., overt domination, suppressing topics, shaping desires, payment) [27, 30, 41–44]. Key attributes of including PWLE in research include early involvement, inclusiveness, co-learning, co-building of knowledge, and providing support [45, 46]. With this knowledge, we wanted a space for AA to collaborate as a member of the research team, providing unique experience-based insight to participants and the VCT. Aware of these challenges and commons models of engagement, the research team integrated several guiding principles reported in the literature [45–49], as well as self-developed principles from briefings. Namely, we used the following five principles to guide our inclusion: autonomy, active voice, flexibility, financial compensation, and ongoing support.

Autonomy was an important component of the research team’s collaboration with AA. She decided on her level of engagement in the study and chose tasks that would complement her interests and skillset. In a qualitative review by Ehrlich et al. [50], the authors explain for there to be successful integration of inter-professional teams, it is essential for clinical staff to understand the unique contributions of PWLE in healthcare settings. AA was highly encouraged to voice her opinions and ideas during case reviews. Reinforcing that her opinion was equally as valuable, as the other healthcare professionals, helped affirm her expertise through experience, thus attempting to decrease power imbalances between AA and the team members. AA had flexibility in her schedule (e.g., evening meetings and flexible deadlines) and could work remotely. AA was compensated (i.e., $30 for the first hour, and $25 for subsequent hours) for her time and expertise at a rate that was recommended by CAMH. Lastly, ongoing support was provided to AA via virtual and in-person check-ins with one of the research team members, as needed. During the check-ins, AA would receive feedback on her involvement and was encouraged to discuss her workload, collaboratively create new tasks for herself, reflect on her involvement and relationships with team members, report any challenges, and express her vision for the project. This level of support allowed the research team to deepen the relationships between team members, leading to better collaboration.

### Experiences and perspectives

By employing the five principles of autonomy, active voice, flexibility, financial compensation, and ongoing support, AA was able to utilize her skills and work within her interests. Overall, AA brought different perspectives providing optimal patient care to both the research team and VCT. The voices of AA, the research team, and VCT came together to create a true collaborative healthcare approach. The research team learned the importance of including a PWLE in the design and development of the project. AA used her expertise to review and provide feedback on the design and development of the e-platform, the main virtual hub where all participants would access educational materials and virtual calls with their health coaches. For example, she ensured that the language and representation of the e-platform used appropriate language to promote a safe environment (e.g., avoiding stigmatizing words). As such, developing an e-platform for youth with psychosis without the input of a PWLE may have caused discomfort and disinterest in participating among participants. The challenges were no different than any other research team when finding a balance between meeting REB deadlines and including input from a diverse set of opinions. The risk of delay in the project is real and must be accounted for apriori in any planning.

Through AA’s contribution to the individualized treatment plans, the VCT was able to apply a holistic lens to healthcare. By referencing the biopsychosocial plus approach to health care [51], AA challenged the VCT to consider all aspects of the participants’ lives, including environmental and social factors. Additionally, AA expressed the importance of a humanistic approach and promoting participant autonomy in health care decisions to increase their level of engagement in their treatment. For example, when a participant expressed a desire to stop their medication, AA reminded the VCT that it is essential to phrase our safety recommendations in a way that does not take away the participant’s autonomy. For the VCT, one challenge included looking beyond the medical lens to understand the individual experiences of the participants. With AA’s contributions, the research team was able to ensure to use the lens of the participant—an essential component to client-centered and holistic care. Further experiences from the research team will be provided in a future publication on the qualitative evaluation component of this study.

Through AA’s involvement on the VCT, clinicians were able to reflect on any underlying assumptions they might have made about participants. This was successfully depicted when members of the VCT suggested the possibility of signs of hypermania for a participant who expressed very ambitious goals. During that discussion, AA had asked the healthcare professionals to consider the possibility that the participant was reverting back to their “previous ambitious self” (i.e., prior to being diagnosed with a mental health illness) rather than showcasing signs of symptom flare-up. For the VCT, it was imperative yet challenging to slow down the anticipated treatment trajectory and shift gears to focus more on building trust with the participants, and understanding them through a humanistic approach. AA’s expertise by experience allowed the VCT to question their own assumptions, allowing way for other perspectives and understandings to influence further treatment planning.

Given the two distinct roles that AA performed, it provided a unique opportunity for a PWLE to be actively involved in all aspects of a research study. At the beginning of AA’s involvement, there was more focus on research-related tasks to begin the study. After recruitment, AA’s role was entirely VCT-related. We found it helpful to separate AA’s timelines for research and VCT-related tasks, in order to manage her workload. The research team found it imperative to regularly check-in (bi-weekly or monthly at minimum) with AA on her tasks for either role, if needed. Overall, the duality of AA’s roles brought many strengths to the study—including increased collaboration between the research team and VCT, continuous patient-perspectives in all study components, and ongoing learning opportunities for researchers, clinicians and PWLE.

### Narrative (Augustina)

In 2012, I went through a period of psychosis and it was the scariest time of my life. After going through an Early Psychosis Intervention program, dancing and doing my own art therapy, I vowed that I would do whatever I could to change the stigma surrounding mental health. At first, I was very afraid of the stigma that I would encounter by telling my story. But during therapy, I felt my heart telling me that I needed to share my story with others in the community. I then started attending a variety of different conferences and telling my story to clinicians, nurses and other mental health advocates. When I was approached by the TECC-Y team to become a team member, I was thrilled. Not only was I able to talk about my own lived experience with mental health throughout the project, but I was able to use my own lived experience to help inform care decisions for other participants. I was able to take on a unique role, I was part of the research team and a clinical team (VCT). This was very important to me because I wanted to do more within the mental health community. On average, I spent 6–8 h monthly in my role. I was able to juggle this role with my full-time job. My primary employer was very supportive and understood my passion for mental health. If there were any scheduling conflicts I was able to inform the team and they were able to accommodate by sending me the meeting minutes and updating me on anything I may have missed.

When I started my role on the VCT, I was nervous and afraid that my voice would not hold as much weight as the others on the VCT given the power dynamic that often happens in research and healthcare teams, however that was never the case. Throughout the project, I was always encouraged and constantly provided my feedback and advice on client cases in weekly VCT meetings. I suggested different ways the participants could engage in self-reflection such as journaling or meditation. This really helped me feel like an equal partner on the team. I not only felt as though I was a valuable team member, but that I was contributing to the mental health community. For PWLE working on care teams, I think it is important for them to be heard and for clinicians to work with them. This will help prevent challenges in collaborating together. In my research role as a PWLE, I provided suggestions on the research protocol, tested the e-platform, moderated a discussion forum for participants, and provided participant webinars. Conducting the webinars was a great experience because I felt that I could connect with the participants and share my story. It was amazing hearing their journeys with psychosis and the questions they had for me. I also attended a research conference to talk about the study. It is important for researchers who want to include PWLE on their research team to engage them early in the development of the project. By having me on the team as a PWLE, I believe I was able to provide unique perspectives and recommendations based on experience. Overall, I believe the collaborative care model is very important because it brings a variety of different perspectives to the table, which allows for innovative ideas for care which can help with better patient outcomes. I think this is something that is currently missing in the healthcare system today for youth with early psychosis, a collaborative approach to recovery.

Although I felt comfortable enough to share my feedback with the team, other PWLE may not. Some may feel too shy or nervous when giving suggestions to the team. It is important to acknowledge that I am an outspoken person naturally (which is why I love doing speeches), therefore I was very comfortable in sharing my experiences. I encourage research teams to include PWLE and ensure that they provide safe environments for healthy discussions. This can be achieved by actively asking the PWLE if they have any thoughts or suggestions that they would like to express regularly during meetings or encourage them to send feedback after the meeting. Encouraging continuous feedback can help the PWLE feel more engaged in the project. Also, while I personally did not encounter any major challenges, it may be difficult for a PWLE with no research experience to understand the inner workings of a research study. It would be best for those individuals to take a few free research courses such as research methods or ethics so they can learn some of the guidelines. It would also be beneficial for the team to explain frequently used acronyms. Lastly, I think it is important to pay PWLE when they are involved in research studies. I was hired and I was provided with an honorarium for participating in this study which was greatly appreciated. It shows appreciation for the time spent on the project.

As such, I strongly encourage PWLE to get involved in research projects if the opportunity presents itself as our voices are strong and powerful. Always remember, you cannot change or improve the system without the voices of PWLE.

## Discussion

Research studies and professional organizations have provided guiding principles and other recommendations for including PWLE in research studies. Nevertheless, similar recommendations on including PWLE in clinical settings are less extensive. In our descriptive paper, we described how we went beyond the initial recommendations and created additional guiding principles in hopes to create an open, effective, and productive working relationship between the PWLE, VCT, and the research team. It was imperative that our research team ensured the experiences of PWLE were relevant and essential to both the research team and the participants, collaborative and productive, and that the interests and preferences among all members of the research team were aligned. Among all our principles, our research team heavily focused on providing autonomy to our PWLE. We found this to be an essential step to create a foundation for trust, collaboration and purposeful work. Other models of PWLE engagement emphasize the importance of collaboration, reciprocal learning and ensuring rewarding experiences [45, 52, 53]. However, to our knowledge, our paper is the first to describe how to engage a PWLE of a mental illness in a clinical component of a research study, with a specific emphasis on providing autonomy and encouraging an active voice.

Our paper is not without limitations. The views expressed by AA is based on their sole experience in the research study, therefore, their opinions of our guiding principles should not be generalized to all PWLE involved in research projects. Additionally, AA had a previous background in research and the healthcare sector, which may have provided an advantage to managing the workload and tasks of the two distinct roles in this study. As such, for future researchers involving PWLE in research, it would be beneficial to provide some research training for the PWLE for enhanced understanding of their research role and more effective collaboration. More frequent support and check-ins may be warranted for PWLE with less research experience.

## Conclusions

By including a PWLE in our study, the research team and VCT were encouraged to adapt a holistic and client-centered model of collaborative care. Our learnings from our PWLE’s personal experiences shaped the way our team viewed research and treatment planning, and we learned to look beyond traditional medical-based lenses to take biopsychosocial plus factors into consideration. We hope that current and future scientists, researchers, and healthcare professionals follow alongside our journey of including PWLE in all aspects of mental health policy, treatment and intervention, to strengthen collaborative care.

## Data Availability

Not applicable.

## Abbreviations

PWLE: People/person with lived experience
CCM: Collaborative Care Model
VCT: Virtual Care Team
TECC-Y: Technology-Enabled Collaborative Care for Youth
